# The effects of cues on neurons in the basal ganglia in Parkinson's disease

**DOI:** 10.3389/fnint.2012.00040

**Published:** 2012-07-26

**Authors:** Sridevi V. Sarma, Ming L. Cheng, Uri Eden, Ziv Williams, Emery N. Brown, Emad Eskandar

**Affiliations:** ^1^Biomedical Engineering Department, Institute for Computational Medicine, Johns Hopkins University, BaltimoreMD, USA; ^2^Department of Neurosurgery, Center for Neurorestoration, Alpert Medical School at Brown University, ProvidenceRI, USA; ^3^Department of Mathematics and Statistics, Boston University, BostonMA, USA; ^4^Department of Neurosurgery, Al-Rohdan Laboratories – MGH-HMS Center for Nervous System Repair, Massachusetts General Hospital, BostonMA, USA; ^5^Division of Health Sciences and Technology, Harvard Medical School/Massachusetts Institute of Technology, CambridgeMA, USA; ^6^Department of Brain and Cognitive Sciences, Massachusetts Institute of Technology, CambridgeMA, USA

**Keywords:** neuron, neuropathology, Parkinson disease, neuromodulation, cueing

## Abstract

Visual cues open a unique window to the understanding of Parkinson's disease (PD). These cues can temporarily but dramatically improve PD motor symptoms. Although details are unclear, cues are believed to suppress pathological basal ganglia (BG) activity through activation of corticostriatal pathways. In this study, we investigated human BG neurophysiology under different cued conditions. We evaluated bursting, 10–30 Hz oscillations (OSCs), and directional tuning (DT) dynamics in the subthalamic nucleus (STN) activity while seven patients executed a two-step motor task. In the first step (predicted +cue), the patient moved to a target when prompted by a visual go cue that appeared 100% of the time. Here, the timing of the cue is predictable and the cue serves an external trigger to execute a motor plan. In the second step, the cue appeared randomly 50% of the time, and the patient had to move to the same target as in the first step. When it appeared (unpredicted +cue), the motor plan was to be triggered by the cue, but its timing was not predictable. When the cue failed to appear (unpredicted −cue), the motor plan was triggered by the absence of the visual cue. We found that during predicted +cue and unpredicted −cue trials, OSCs significantly decreased and DT significantly increased above baseline, though these modulations occurred an average of 640 ms later in unpredicted −cue trials. Movement and reaction times were comparable in these trials. During unpredicted +cue trials, OSCs, and DT failed to modulate though bursting significantly decreased after movement. Correspondingly, movement performance deteriorated. These findings suggest that during motor planning either a predictably timed external cue or an internally generated cue (generated by the absence of a cue) trigger the execution of a motor plan in premotor cortex, whose increased activation then suppresses pathological activity in STN through direct pathways, leading to motor facilitation in PD.

## Introduction

An estimated 6.5 million people world-wide have Parkinson disease (PD), a chronic progressive neurological disorder that occurs when dopaminergic neurons in the midbrain degenerate, causing motor deficits including tremor, rigidity, and bradykinesia (Lang and Lozano, 1998a,b). An intriguing aspect of PD is the dynamic nature of these motor symptoms. Clinical observations show that tremor is attenuated with movement (Lang and Lozano, 1998b) while visual and auditory cues improve gait (Georgiou et al., 1993; Morris et al., 1996; Azulay et al., 1996, 1999; Suteerawattananon, 2004), movement velocity, movement accuracy (Majsak et al., 1998), reaction times (Kühn et al., 2004), and off freezing (Kompoliti et al., 2000). There are different proposals regarding the mechanism underlying cue-related improvements in motor function. One hypothesis is that alternative preserved visual-motor pathways bypassing the basal ganglia (BG) facilitate motion responsiveness to visual cues (Glickstein and Stein, 1991). Another hypothesis is that increased cortical drive associated with cues leads to transient dampening of pathological 10–30 Hz oscillations in the BG, which in turn facilitates movement (Amirnovin et al., 2004).

Previous independent works suggest that in PD patients, (1) cues lead to increased cortical activity in premotor and motor areas which facilitates movement and (2) cues lead to suppression of pathological activity in the subthalamic nucleus (STN) of the BG which facilitates movement. Jahanshahi et al. (1995) showed increased cortical activity (when compared to rest) in cortical regions when PD patients either self-initiated movements or moved in response to a tone presented at a predicted rate. Kühn et al. (2004) showed that pathological 10–30 Hz oscillations (OSCs) in local field potentials from the STN region of PD patients decreased immediately after a cue to move was presented, with an onset latency that strongly correlated with mean reaction times. These results are consistent with findings that in PD patients, beta oscillations in single-unit recordings of STN neurons were suppressed during visually guided movement (Amirnovin et al., 2004) and active voluntary movement (Levy et al., 2002). Taken together, the literature may suggest that in PD patients cue-driven cortical activity is responsible for decreasing pathological BG activity that facilitates motor function.

It is important that the cortical activation reported in (Jahanshahi et al., 1995) occurred when patients could either predict the timing of the cue or when they self-initiated movements. Two important questions that we ask here are (1) “what happens when the timing of cues cannot be predicted by patients?” and (2) “what if an expected cue never appears forcing the patient to move in the absence of a cue?” In the same study, Jahanshahi showed that when patients could not predict cue timing, reaction times increased and there was no significant increase in cortical activity.

We set out to answer these two questions with respect to BG neurophysiology and movement-related behavior in PD patients. Our hypothesis was that the inability to predict cue timing would diminish dampening of pathological BG activity observed when cues are presented in a predictable fashion. We compared the effects of predictable and unpredictable cues on behavior and spiking activity in 28 STN neurons recorded from seven PD patients executing a two-step center-out task during deep brain stimulation surgery.

## Materials and methods

### Subjects

Seven patients undergoing deep brain stimulator placement for the treatment of PD were included in the study. All patients had idiopathic PD with a Hoehn–Yahr score of three or higher and had a documented response to L-dopa replacement therapy. All patients received a thorough pre-operative neurological exam. Exclusion criteria for surgery included those patients with Parkinson “plus” syndromes, cognitive impairment, active psychiatric disorders, or anatomic abnormalities on magnetic resonance imaging (MRI) (Amirnovin et al., 2006). None of the patients had undergone prior surgery for the treatment of PD. Informed consent for the study was obtained in strict accordance with a protocol approved by the Institutional Review Board and the multidisciplinary movement disorders assessment committee at the Massachusetts General Hospital. The decision to offer surgery was based on clinical indications alone, and bore no relation to the patients' participation in this study. To ensure that the patients were comfortable with performing the behavioral joy-stick task, they practiced the task prior to the surgery until they reached 90% success or more on all trial types. Subjects were able to remove their hand from the joystick or stop the task at any time. At all-time points before and during surgery, the patients had the clear understanding that their participation was not related to the surgical outcome, and that they could withdraw from the study at any time.

### Electrophysiology

Anti-Parkinsonian medications were withheld the night before surgery. No sedatives were given prior to or during performance of recordings. A local anesthetic was used prior to the incision and burr hole placement. The stereotactic localization using pre-operative MRI and computerized tomography, as well as general techniques of intraoperative microelectrode recordings have been described previously (Abosch et al., 2002; Amirnovin et al., 2006). Single-unit recordings were made from the dorsal-lateral motor sub-territory of the STN based on stereotactic localization and reconstructions of the electrode trajectories (Abosch et al., 2002). The STN has characteristic high firing rates in comparison to the surrounding structures (Hutchison et al., 1998) and has clear dorsal and ventral borders that are evident when reconstructing neuronal activity along the electrode trajectories. Once within the STN, no attempt was made to explicitly select cells based on presence or absence of movement-related activity, or on whether the cells responded to passive and/or volitional movement. This was done specifically to limit the potential for a sampling bias.

We used an array of 3 tungsten microelectrodes, separated by 2 mm and placed in a parasagittal orientation. The electrodes were advanced simultaneously in 50-μ increments using a motorized micro-drive (Alpha Omega; Nazareth, Israel). The behavioral paradigm was controlled by a Macintosh G4 computer using custom-made software. Neuronal activity was band-pass filtered (300 Hz–6 kHz) and sampled at 20 kHz. Spikes were sorted off-line using a standardized template-matching algorithm (Cambridge Electronics Design, Cambridge, England).

### Behavioral task

**Figure 1:**
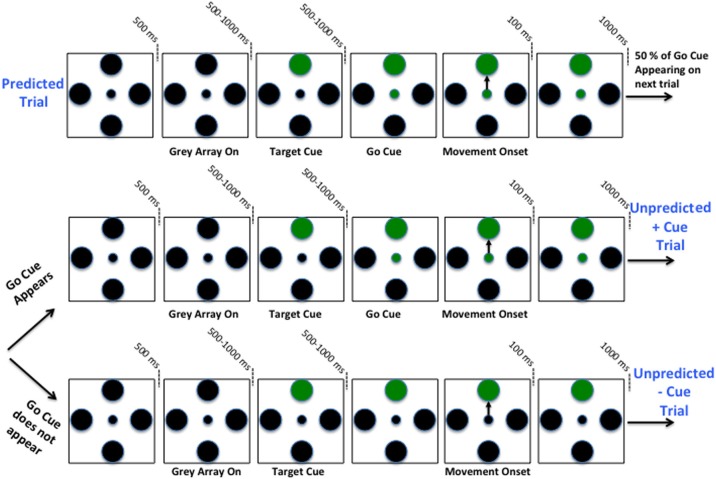
**Schematic of two-step sequential motor task.** Predicted +Cue trial (Top), Unpredicted +Cue trial (middle), Unpredicted −Cue trial (bottom).

Once the microelectrodes were in the STN and stable single units were obtained, the subjects viewed a computer monitor and performed a two-step behavioral task by moving a joystick (mounted such that movements were in a horizontal orientation with the elbow flexed at approximately 45°) with the contra-lateral hand. Each task pair consisted of a *predictable cue trial* followed by an *unpredictable trial*. Refer to Figure 1. In the predictable cue trial, a central fixation spot (0.2° in diameter) was first displayed for 500 ms, after which, an array of four gray equally spaced circular targets (1° in diameter) would appear in the “up”, “right”, “down”, and “left” directions. After a variable delay interval ranging 500–1000 ms, one of the gray targets would turn green. Following another variable delay interval ranging from 500 to 1000 ms, the central fixation spot would turn green indicating that the patients could move the joystick. Once within the target, patients were required to hold the cursor stationary for another 100 ms. The stimuli on the screen would then erase, and the patients would be allowed to return the spring-loaded joystick to its resting position. It is important to note that patients knew a priori that a go cue will appear during these trials within a predictable window of time.

After completion of the first-step trial, the screen remained blank for 1000 ms. This would be followed by one of two possible unpredictable trials. As before, a fixation spot and gray circular array would appear, but would be followed by a go cue with 50% chance. In these trials, the patients were required to move the joystick in the same direction as in the preceding trial. If the patient moved and a go cue appeared afterwards or if the patient waited too long before moving (≥4 s after fixation), he/she would not complete the second-step trial successfully. Therefore, patients had an incentive to wait for a possible go cue and if no go cue appeared within a short time period then the *lack of the cue* triggered movement. In fact, after 1 s following the presentation of a target cue, if the go cue does not appear, then with certainty it will not be coming and subjects should self-initiate the movement. Patients had to learn this over time, and when each trial was completed, a sound tone was generated indicating whether or not they completed it correctly.

It is important to note that the predictable trials and the unpredictable trials in which the go cue is presented are identical, with the exception that *the timing* of the go-cue in the latter is not as predictable. The trials were pseudo-randomly interleaved in blocks such that each direction and trial type was presented once within each block, rendering a 50% chance of a go cue appearing on any given unpredicted trial. All directions and trial types were counterbalanced such that an equal number of directions and trials types were tested for each cell. Furthermore, variable delays for cue presentation on +cue trials were each timed separately. If patients strayed beyond the confines of a 60°-wide invisible corridor, moved the cursor to an incorrect target, failed to return the joystick to its resting position or failed to reach the target within 4 s from fixation, the trial would abort and repeat again. The patients were instructed to maintain their gaze on the center of the screen at all time-points during the trial. See Figure 1 for a schematic of two-step sequential motor task.

Table 1 below itemizes the distribution of trials and neurons recorded per patient that contributed to the models used for our analysis. As described below, the neurons used in the analysis gave rise to point process models that met a goodness-of-fit criterion.

**Table 1:** **Distribution of trials and recorded neurons per patient**.

**Patient ID**	**Number of 2-step paired trials executed**	**Total number of neurons recorded**	**Number of neurons included in analysis (Anticipated-cued trials)**	**Number of neurons included in analysis (Un-anticipated-cued-trials, visually-guided)**	**Number of neurons included in analysis (Un-anticipated-cued-trials, self-initiated)**
1	159	5	4	4	4
2	38	3	3	3	1
3	141	6	6	6	6
4	24	7	2	2	2
5	44	4	2	2	2
6	189	7	7	4	2
7	100	5	4	4	4
Total	695	37	28	25	21

### Statistical analysis: point process models of STN dynamics

We analyze neuronal spiking activity in STN neurons by constructing point process models (Barbieri et al., 2001; Brown et al., 2001a,b; Truccolo et al., 2005, 2010). The point process framework has proven in practice to be a powerful and flexible framework that is capable of modeling spike train activity from a diverse range of neuronal types and neural circuits, such as: place cells from the rat hippocampus (Barbieri et al., 2001); retinal ganglion cells of the salamander, rabbit, and cat (Keat et al., 2001); neurons from the supplementary eye field of the macaque monkey (Kass and Ventura, 2001); and STN neurons of PD patients (Levy et al., 2001; Paninski, 2004; Eden et al., 2007; Czanner et al., 2008; Montgomery, 2008; Zelnikera et al., 2008; Sarma et al., 2010).

A point process model of a single neuron can capture the relative contribution of short and long-term history effects (temporal dependencies), movement direction, and the impact of external cues on the probability that the neuron will spike at any given time. Since STN neurons in PD patients exhibit pathological oscillations (Hutchison et al., 1994; Bergman et al., 1994; Levy et al., 2000; Brown et al., 2001a,b; Dostrovsky and Bergman, 2004; Gale et al., 2009; Sarma et al., 2010 and more), the short and long-term history effects become significant factors on spiking probabilities. In addition, PD STN neurons exhibit increased directional tuning (DT) after movement (Crutcher and DeLong, 1984; Williams et al., 2005; Sarma et al., 2010), therefore movement direction will influence the models. Finally, since external cues such as visual cues and movement onset play a significant role in altering behavior in PD patients, it is likely that these extrinsic factors will also impact neuronal spiking probabilities of STN neurons. The point process framework thus enables us to study the dynamics of all characteristics (bursting, oscillations, and DT) in STN spiking activity simultaneously in an efficient and statistically sound manner.

A point process is a series of 0–1 random events that occur in continuous time. For a neural spike train, the 1 s is individual spike times and the 0 s are the times at which no spikes occur. To define a point process model of neural spiking activity, in this analysis we consider an observation interval (0, *T*], and let *N*(*t*) be the number of spikes counted in interval (0, *T*] for *t* ∈ (0, *T*]. A point process model of a neural spike train can be completely characterized by its conditional intensity function (CIF), λ(*t* | *H*_t_), defined as

(1) $$\lambda (t|H_{t})=\underset{\Delta \to \text{0}}{\lim}\frac{\mathrm{Pr}(N(t+\Delta )-N(t)=1|H_{t})}{\Delta }$$

where *H*_*t*_ denotes the history of spikes up to time *t*. It follows from (1) that the probability of a single spike in a small interval (*t*, *t*+Δ] is approximately

(2) $$\mathrm{Pr}(\text{spike in}(t,\;t+\Delta ]|H_{t})≅\lambda (t|H_{t})\Delta .$$

When Δ is small, Equation (2) is roughly the spiking propensity at any time *t* (Daley and Vere-Jones, 2003; Snyder and Miller, 1991). The well-known homogeneous Poisson process is a special point process in which all events are independent and the CIF does not dependent on history. Because the CIF characterizes a point process in its entirety, defining a model for a CIF defines a model for the spike train.

We use generalized linear models (GLMs; Truccolo et al., 2005) to characterize the CIF for each neuron. In a GLM, the log of the CIF is a modeled as a linear function of parameters that multiply the covariates which describe the neural activity dependencies The GLM is an extension of the multiple linear regression model in which the variable being predicted, in this case spike times, need not be Gaussian (McCullagh and Nelder, 1989). GLM also provides an efficient computational scheme for model parameter estimation and a likelihood framework for conducting statistical inferences based on the estimated model (Brown et al., 2003).

**Figure 2:**
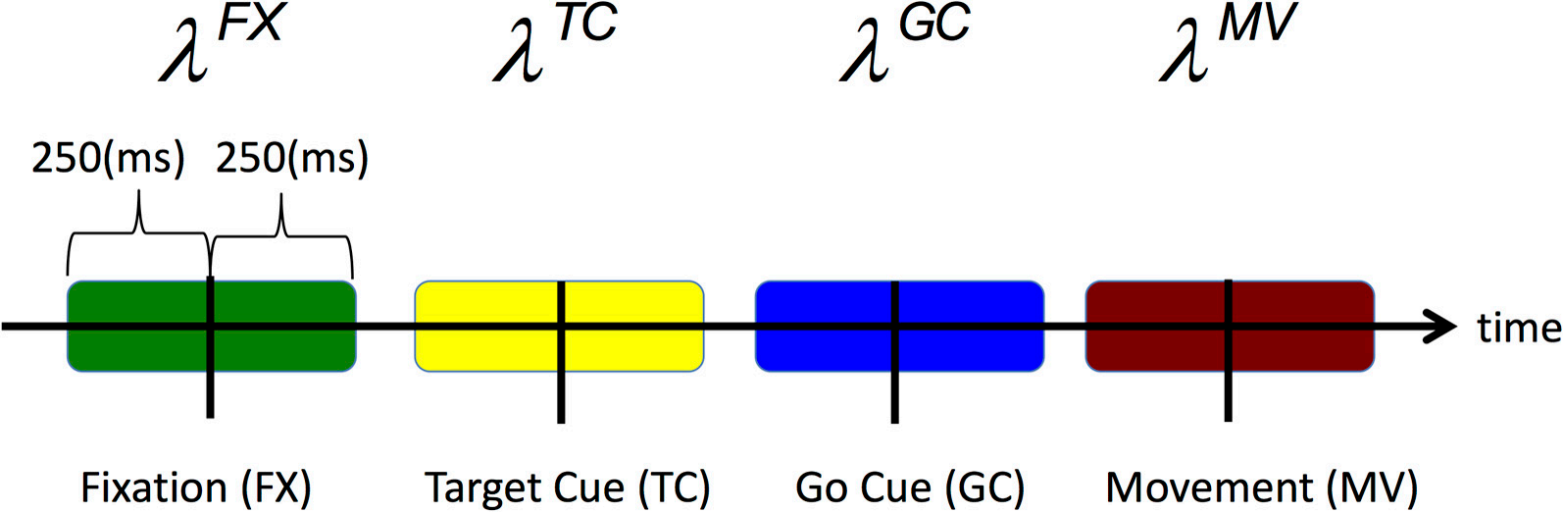
**Time periods over which the CIF denoted by Equation (3), is estimated are shaded**.

For each trial type, we define the CIF for each neuron to be a function of movement direction *d* ∈ {1, 2, 3, 4} which corresponds to {Up, Right, Left, and Down} and the neuron's spiking history in the preceding 150 ms. Rather than estimating the CIF continuously throughout the entire trial, we estimate it over specific time windows around key epochs and at discrete time intervals each 1 ms in duration. In particular, we estimate the CIF over 500 ms during fixation (FX) and over 250 ms windows centered at the target cue onset (TC), go cue onset (GC), and movement onset (MV) onsets. Figure 2 highlights all of the time periods for which we estimate the CIF for each neuron.

We omit the superscripts denoting the epoch for a simpler read and define the rate function as

(3) $$\lambda (t|H_{t},\;\theta )=\lambda^{s}(t|H_{t},\;\theta )\;\cdot \;\lambda^{H}(t|H_{t},\;\theta )$$

where the component λ^*s*^(*t*|*H*_*t*_, θ) describes the effect of the behavioral stimulus (movement direction) on the neural response and the component λ^*H*^(*t*|*H*_*t*_, θ) describes the effect of spiking history on the neural response. θ ={α, β, γ} is a parameter vector to be estimated from data and is defined below. The units of λ (*t* | *H*_*t*_, θ) and λ^*s*^(*t* | *H*_*t*_, θ) are in spikes per second and λ^*H*^(*t*|*H*_*t*_, θ) is dimensionless. The idea to express the CIF as a product of a stimulus component and a temporal or spike history component was first suggested by (Kass and Ventura, 2001) and is appealing as it allows one to assess how much each component contributes to the spiking propensity of the neuron. If spiking history is not a factor associated with neural response, then λ^*H*^(*t* | *H*_*t*_, θ) will be very close to 1 for all times and Equation (3) becomes an inhomogeneous Poisson process.

Our model of the stimulus effect, which depends on the movement direction, is

(4) $$\log\lambda^{S}(t|d,\;\alpha )=\alpha_{d}$$

The α = {α_*d*_}^4^_*d* = 1_ parameters measure the effects of movement direction on the spiking probability. Our convention is *d* = {1, 2, 3, 4} = {Up, Right, Down, Left}. For example, if *e*^α_1_^ is significantly larger than *e*^α_2_^, *e*^α_3_^, and *e*^α_4_^ during movement, then the probability that a neuron will spike (λ · Δ), in small time interval Δ is greater when the patient moves in the “Up” direction, suggesting that the neuron may be tuned in the “Up” direction.

Our model of the spike history effect is

(5) $$\log\lambda \left(t|H_{t},\;\beta \text{, }\gamma \right)=\sum_{i=1}^{10}\beta_{i}n_{t\text{-}j:t\text{-}(j\text{+1})}+\sum_{j\text{=1}}^{\text{14}}\gamma_{j}n_{t\text{-}(\text{10}j\text{+9}):t-10j}$$

where *n*_*a:b*_ is the number of spikes observed in the time interval (*a, b*] during the epoch.

The {β_*j*_}^10^_*j* = 1_ parameters measure the effects of spiking history in the previous 10 ms and, therefore, can capture refractoriness and/or bursting on the spiking probability in the given epoch. For example, if *e*^β_1_^ is close to zero for any given epoch, then for any given time *t*, if the neuron had a spike in the previous millisecond then the probability that it will spike again is also close to zero (due to refractory period). Or if *e*^β_5_^ is significantly larger than 1, then during fixation and for any time *t*, if the neuron had a spike 5 ms ago then the probability that it will spike again is modulated up, suggesting bursting.

The {γ_*j*_}^14^_*j* = 1_ parameters measure the effects of the spiking history in the previous 10–150 ms on the spiking probability, which may be associated with not only the neuron's individual spiking activity but also that of its local neural network. For example, if *e*^γ_4_^ is significantly larger than 1, then for any time *t* during fixation if the neuron had one or more spikes between 40 and 50 ms ago then the probability that it will spike again is modulated up, suggesting 20–25 Hz oscillations. We describe how we used the model parameters to quantify these spiking characteristics in detail in the Results section.

By combining Equations (3), (4), and (5), we see that the CIF GLM for a given neuron may be written as

(6) $$\log\lambda (t|d,\;H_{t},\;\theta )=\alpha_{d}+\sum_{i=1}^{10}\beta_{i}n_{t-j:t-(j\;+1)}+\sum_{j=1}^{14}\;\gamma_{j}n_{t-(10j+9):t-10j}$$

The model parameter vector θ = {α, β, γ} contains 28 unknown parameters (for each epoch and for each time window modeled). We computed maximum-likelihood estimates for θ and 95% confidence intervals of θ for each neuron using glmfit.m in MATLAB (Brown et al., 2003). We also used the Kolmogorov–Smirov (KS) statistic, based on the time-rescaling theorem, to assess model goodness-of-fit (Brown et al., 2002; Truccolo et al., 2005).

Finally, it is important to note that building a point process model of spiking activity of a neuron is equivalent to estimating the joint distribution function for the random spiking process (Sarma et al., 2010). If the estimate of this distribution is satisfactory, then any first and second order statistic (e.g., inter-spike interval histogram, spectrogram, tuning vector, etc.) can be computed using simulated data from the estimated distribution. That is, the point process model encompasses any traditional statistic used to analyze bursting, oscillations or DT in spiking data. Furthermore, traditional statistics can lead to erroneous inferences as shown in more detail in (Sarma et al., 2010).

### Determining spike train characteristics from point process models

Recall from (2) that the product of the rate function for a given neuron and a small time interval, λ(*t*|*H*_*t*_, θ) · Δ, is approximately the probability that the neuron will fire in time interval (*t*, *t* + Δ] given history of extrinsic and intrinsic dynamics up to time *t*, which is captured in *H*_*t*_. Then by virtue of Equations (2) and (6), we allow the probability that each STN neuron will spike at some time *t* within an epoch (*ep*) to be modulated by movement direction (captured in {α_*d*_^*ep*^}_*d* = 1_^4^ parameters), short-term history spiking dynamics (captured in {β_*j*_^*ep*^}_*j* = 1_^10^ parameters) and long-term history spiking dynamics (captured in {γ_*k*_^*ep*^}_*k*_ = 1^14^ parameters).

**Figure 3:**
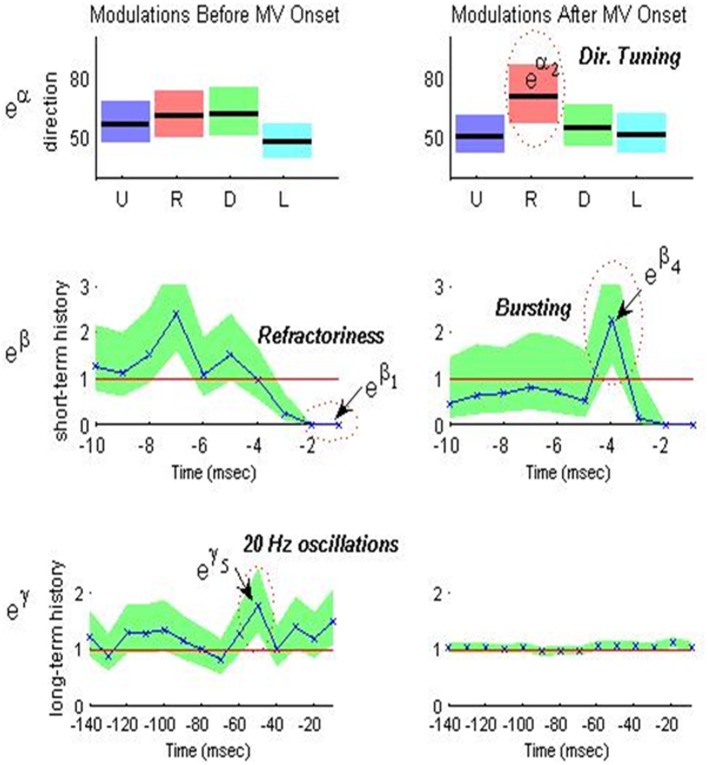
**Optimal model parameters for an STN neuron during MV – and MV+ periods of a PD patient executing predicted-cued trials before movement (left) and after movement (right).** Top row (movement direction modulation): optimal extrinsic factors *e*^α_*d*_^ for *d* = 1, 2, 3, 4 (U,R,D,L) are plotted in black lines from left to right and corresponding 95% confidence intervals are shaded around each black line in a unique color for each direction. Middle row (short-term history modulation): optimal short-term history factors *e*^β_*i*_^ for *i* = 1, 2,…, 10 are plotted in blue from right to left and the corresponding 95% confidence intervals are shaded in green. Bottom row (long-term history modulation) optimal long-term history factors *e*^γ_*j*_^ for *j* = 1, 2,…, 14 are plotted in blue from right to left and corresponding 95% confidence intervals are shaded in green. Note the change in time scale for bottom row.

Figure 3 shows an example of a single neuron's optimal model parameters and their 95% confidence intervals during the peri-movement epoch. We highlight in Figure 3 and discuss below how certain parameter value ranges indicate refractoriness, bursting, OSCs, and DT.

1. ***Refractoriness:*** As illustrated in the second row of Figure 3, the PD STN neuron exhibits refractory periods as indicated by down modulation by a factor of 10 or more due to a spike occurring 1 ms prior to a given time *t*. That is, if a spike occurs 1 ms prior to time *t*, then it is very unlikely that another spike will occur at time *t* (*e*^β_1_^ ≤ 0.1 for all *e*^β_1_^ within its 95% confidence band). Refractoriness is expected since after an action potential (a spike) occurs, as some time (refractory period) must elapse before a neuron can again produce another action potential in response to a stimulus (Brodal, 1998).
2. ***Bursting:*** As illustrated in the second row of Figure 3, the PD STN neuron fires in rapid succession before *and* after movement onset as indicated by one or more of the short-term history parameters (*e*^β^_*i*_ for *i* = 2, 3,…, 10) corresponding to 2–10 ms in the past being larger than 1. That is, if a spike occurs 2–10 ms prior to time *t*, then it is more likely that another spike will occur at time *t*. More formally, define *LB*_*i*_ and *UB*_*i*_ as the 95% lower and upper confidence bounds for *e*^β_*i*_^ such that *LB*_*i*_ ≤ *e*^β_*i*_^ ≤ *UB*_*i*_ for *i* = 2, 3,…, 10. Then, if *LB*_*i*_ > 1 and *UB*_*i*_ > 1.5 for at least one *i* = 2, 3,…, 10, the neuron exhibits bursting.
3. ***10–30 Hz Oscillations (beta):*** As illustrated in the third row of Figure 3, the PD STN neuron exhibits 10–30 Hz oscillatory firing *before* movement. That is, the probability that the PD STN neuron will fire at a given time *t* is modulated up if a spike occurs 30–100 ms prior to *t*. Again, define *LB*_*j*_ and *UB*_*j*_ as the 95% lower and upper confidence bounds for *e*^γ_*j*_^ such that *LB*_*j*_ ≤ *e*^γ_*j*_^ ≤ *UB*_*j*_ for *j* = 3, 4, 5. Then, if *LB*_*j*_ > 1 and *UB*_*j*_ > 1.5 for at least one *j* = 3, 4, 5, the neuron exhibits 10–30 Hz oscillations.
4. ***Directional Tuning:*** As illustrated in the first row of Figure 3, the PD STN neuron appears to exhibit more DT *after* movement onset. That is, the PD neuron seems more likely to fire in one direction more than at least one other direction. To quantify DT, we performed the following test for each neuron and each epoch:
  - For each direction d^*^ = {*U, R, D, L*}, compute p_d^*^,d_ = Prob(*e*^α_d_*^ > *e*^α_*d*_^) = Prob(α_d^*^_ > α_*d*_) for *d* ≠ d^*^. Define *p*_d_^*^, d^*^ =0. Use the Gaussian approximation for α_*d*_, which is one of the asymptotic properties of ML estimates to compute p_d_^*^, d (Brown et al., 2003).
  - If $\underset{d*=1,2,3,4}{\max}$ p_d_^*^, d ≥ 0.975 then neuron exhibits DT.

**Figure 4:**
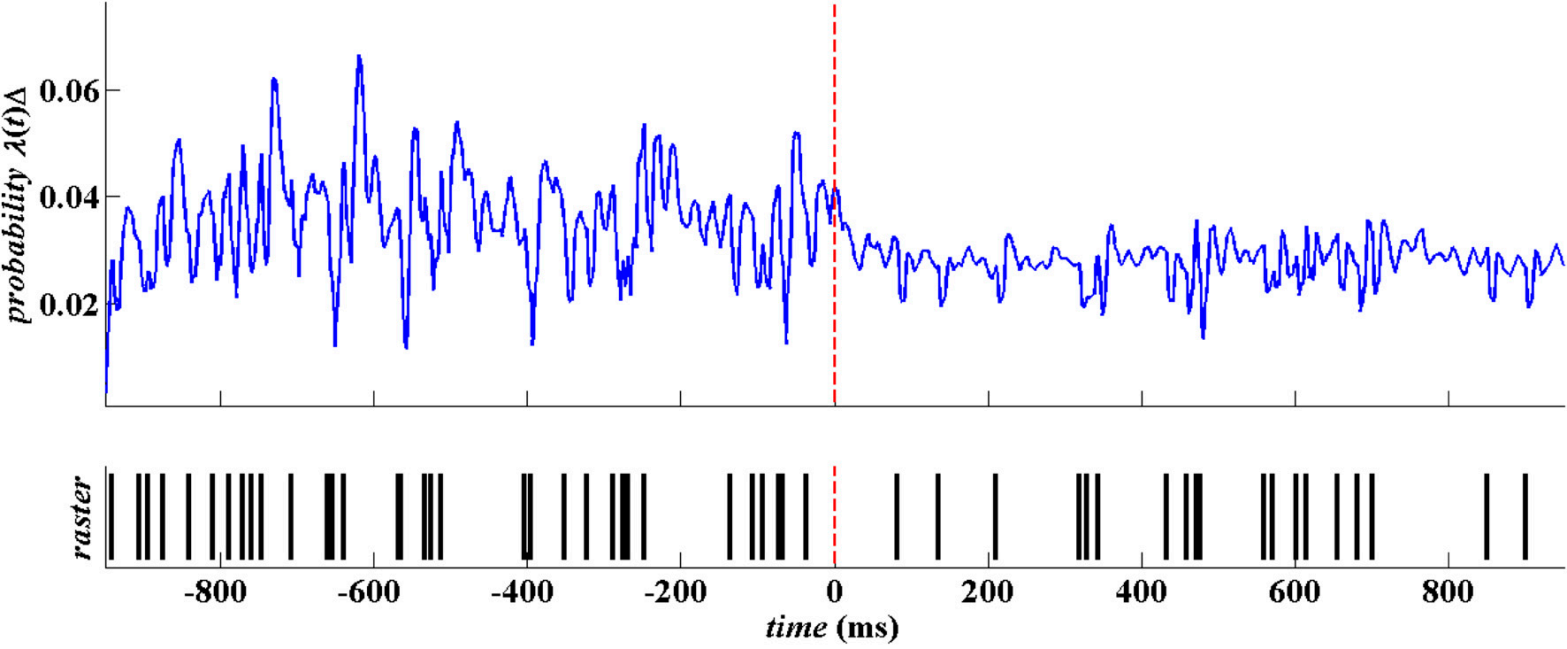
**Raw spike train data from a single STN neuron in PD patient.** The spike train is in blue and the corresponding estimate of the CIF is in red.

Figure 4 below is a snapshots of the CIF estimate from one STN neuron in a PD patient before movement and after movement along with the spike train data. As shown in the top row of Figure 4, the beta oscillations can be seen in the estimate of the CIF itself as the time between the large amplitude peaks are about 40 ms apart which corresponds to a 25 Hz oscillation. These large peaks are attenuated after movement onset (denoted as *t* = 0 in Figure 4).

## Results

### Effects of predictable and unpredictable cues on STN activity

Since we had spike train data for 37 STN neurons across the seven patients (see Table 1), we built point process models for all 37 STN neurons. A total of 28 neuron models passed our goodness-of-fit criterion which required the KS statistic to be within its 95% confidence bounds (Johnson and Kotz, 1970). Using these 28 models, we determined for each neuron and for each epoch within the trial, whether the neuron exhibited refractoriness, bursting, OSCs, and/or DT.

**Figure 5:**
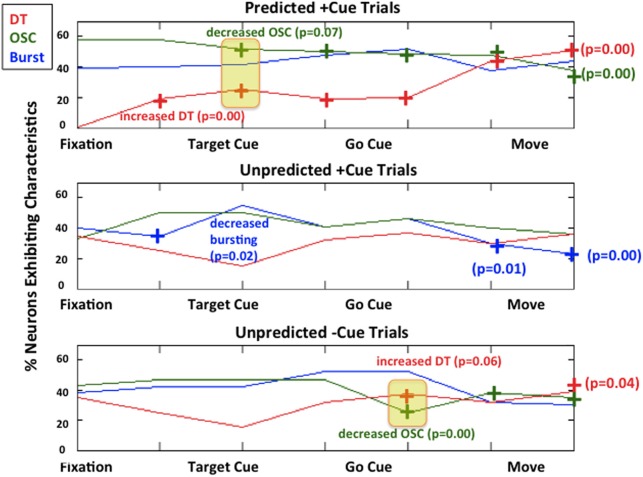
**Modulations of each characteristic for each trial type.** Predicted +Cue Trials (top); Unpredicted +Cue Trials (middle); Unpredicted −Cue Trials (bottom). When the percentage of neurons exhibit neuronal spiking characteristics in a monotonically decreasing less pathological direction (decreasing beta oscillations, increasing directional tuning) for the duration of the trial, we denote that with a “+” symbol.

Figure 5 illustrates a population summary of modulations in bursting, 10–30 Hz oscillations, and DT for each trial type. We do not plot a summary for refractoriness as 100% of the neurons exhibited refractoriness during all epochs in all 3 trial types. When the fractional change from baseline (defined to be the first 500 ms of each trial-fixation or FX) is statistically significant with at least 90% confidence in a less pathological direction (i.e., decreased bursting, decreased OSC, increased DT), we denote it with a “+” symbol and also note the *p*-value. We used standard sign test to look for significant differences from baseline within each trial type because it is a robust test that does not make any assumptions on the distributions of the random variables we are trying to compare (Zar, 1999). In this case, the two random variables we compare for each epoch after fixation within a trial are (1) the percentage of neurons that exhibit a characteristic during the epoch (2) the percentage of neurons that exhibit a characteristic during fixation.

As shown in Figure 5, during predicted +cue trials (top row), there is an increase in DT and a decrease in beta oscillations *early on* during the trial immediately after target cue onset. After movement, this suppression of pathological activity becomes more pronounced, which has been previously reported in studies where patients could predict go cues (Alexander and Crutcher, 1990; Amirnovin et al., 2004; Williams et al., 2005; Gale et al., 2009; Sarma et al., 2010). During unpredicted trials where the absence of a cue triggers movement (Figure 5, bottom row), we also see an increase in DT and a decrease in beta oscillations *later on* during the trial (on average 640 ms after target cue onset).

Interestingly, during unpredicted +cue trials (middle row), we did not observe significant suppression of beta oscillations or significant increase in DT at any time during the trial even though cues were presented.

### Effects of predictable and unpredictable cues on behavior

**Figure 6:**
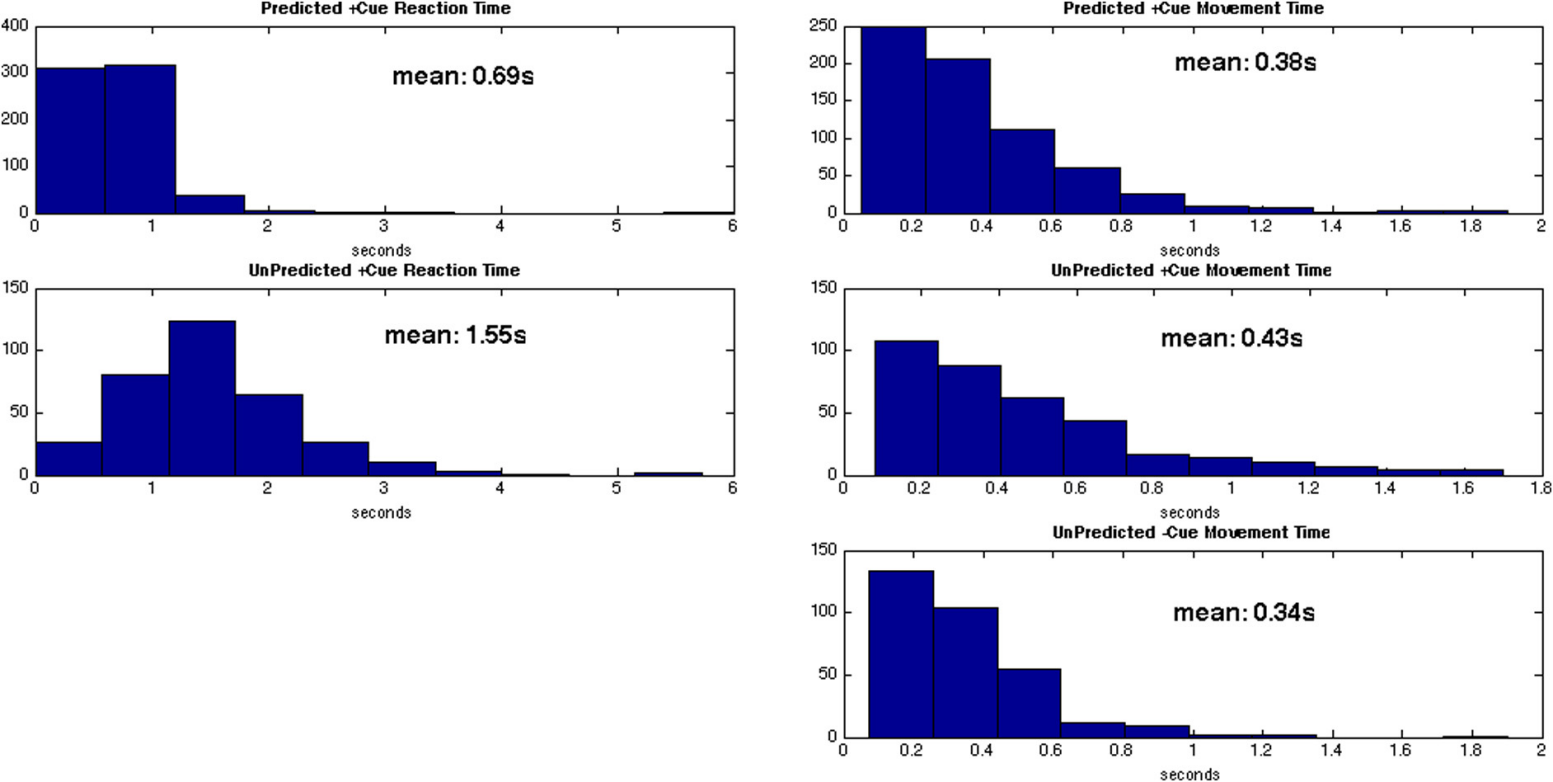
**Distributions for reaction times (left) and movement times (right) for each trial type**.

Distributions of behavior for each trial type are given in Figure 6. The average reaction time for predicted +cue trials is 0.69 s, and the average movement times for predicted +cue trials and self-initiated −cue trials are 0.38 and 0.34 s, respectively. On the other hand, motor performance deteriorated in the unpredicted +cue trials. Specifically, the average movement and reaction times are 0.43 and 1.55 s, respectively.

For behavioral responses, we tested for statistically significant differences between the 3 trial types using a series of two-sample KS test (Johnson and Kotz, 1970). The two-sample KS test checks whether the two data samples come from the same distribution and does not specify what that common distribution is (e.g., normal or not normal). We summarize the results in Table 2.

**Table 2:** **Results for 2 sample KS test for all possible trial pair comparisons**.

**Two trials to compare**	**Behavior variable**	**2 sample KS test results**
Predicted +cue	Reaction times	Reject Null Hypothesis
Unpredicted +cue		*p*-value = 3.63 × 10^−64^
Predicted +cue	Movement times	Reject Null Hypothesis
Unpredicted +cue		*p*-value = 2.72 × 10^−6^
Predicted +cue	Movement times	Accept Null Hypothesis
Unpredicted +cue		*p*-value = 0.1007
Unpredicted +cue	Movement times	Reject Null Hypothesis
Unpredicted +cue		*p*-value = 0.0052

As shown in Table 2, the predicted +cue trials and unpredicted −cue trials do not significantly differ in movement times. In contrast, the predicted +cue trials and the unpredicted +cue trials significantly differ in both movement and reaction times. The unpredicted +cue and unpredicted −cue trials also significantly differ in movement times. These results suggest that behavioral performance is comparable only during predicted +cue and unpredicted −cue trials, which are the only two trial types for which we also observe suppression of pathological neural activity.

## Discussion

To our knowledge, previous works that study the effects of cues on BG neurophysiology in PD patients entail experimental set ups for which cues can be entirely predicted by patients. Two important questions that we ask here, which may shed new light on the underlying mechanisms behind cue-related movements, are (1) “what happens when the timing of cues cannot be predicted by patients?” and (2) “what if an expected cue never appears forcing the patient to move in the absence of a cue?” Specifically, we took a traditional directed hand-movement task and split it into cases where cues can and cannot be predicted before the start of each trial. Our hypothesis was that the inability to predict the timing of a presented external cue would diminish dampening of pathological BG activity observed with cue presentation when cue timing is predictable.

### Effects of timing of external cues

Two of the trial types performed, *predicted* +cue and *unpredicted +cue*, were identical in terms of visuospatial timing and presentation, including the presence of go cues in both (top and middle, Figure 1). The only difference between these two task conditions was the subject's ability to predict the timing of the go cue. This anticipatory difference resulted in increasing suppression of pathological beta oscillations beginning early on during the trial and improvement in reaction time and movement time in the former compared to the latter.

These results suggest that the timing of the external cue must be anticipated to activate a motor plan and effectively trigger movement in PD. There is evidence that premotor cortical areas show increased activity when the timing of an external cue is predictable in patients (Jahanshahi et al., 1995; Paradiso et al., 2003). Since there are direct projections (hyperdirect pathway) to the STN from these cortical areas (Carpenter et al., 1981; Canteras et al., 1990), the firing rates in the STN also show increased activity from baseline (Paradiso et al., 2003). Consequently, the pathological beta oscillations in STN seen in PD may be dampened, perhaps by inactivation of the resurgent sodium current (Do and Bean, 2003). Finally, dampening of these excessive oscillations may facilitate movement.

### Effects of internal cues generated by the absence of expected external cues

In contrast, two of the trial types, the *predicted +cue* and *unpredicted −cue* conditions, resulted in suppression of pathological beta oscillations and improvement in behavioral measures. As we noted, the timing of the suppression occurred later in the *unpredicted −cue* trial type. The suppression of pathological BG oscillations occurred in both trial types despite their visuospatial dissimilarity, with the presence of the go cue in the former and the lack of a go cue in the latter.

In the *unpredicted* −cue condition, our PD subjects were compelled to move by an impending deadline, and movements were triggered in the absence of a cue. There is a 50% chance the external go cue will appear at the start of each trial. If the subject does not move by the end of the go cue epoch, which is defined whether the cue is presented or not, the subject fails the trial and no reward is received. Thus, at some point during the go cue epoch, the subject decides to self-initiate movement in the absence of an external cue. We term this internal impetus to move an “internally generated” cue. Our findings suggest that this internally generated cue is as effective as the external cue in the *predicted* condition in continuously suppressing beta oscillations, increasing DT, and decreasing movement time.

These findings are consistent with those reported in (Jahanshahi et al., 1995) which also showed that premotor cortical areas show increased activity when movements are triggered internally (e.g., self-initiated movements). Therefore, the internal impetus to move may activate prefrontal cortical activity that then triggers the same downstream effects that dampen pathological activity and facilitate movement as do predicted external cues, without requiring the presentation of the external cue.

Although there is no way to determine when the internal cue was generated by the subject, the internal cue should be generated on average after the external go cue would usually have appeared; that is, when the subject realizes that the external cue is not coming and an internal cue is necessary. This leads to the prediction that, if both the internal and the external cues result in PD movement facilitation via the same physiological mechanism, this modulation would occur *earlier* in *predicted +cue trials* than in *unpredicted −cue* trials. Indeed, we find the neurophysiological changes seen in beta oscillations and DT occur on average 640 ms after those seen in *predicted* +cue trials, as we would have predicted.

Why does the *unpredicted +cue* condition result in greater oscillatory activity in the beta band, decreased DT after movement onset, and increased reaction and movement times in comparison to the other two conditions? An “expectation of movement” may be important in both clinical PD behavior and the physiology of a cue-related response. It is well known that cues activate the PD condition. The use of different cue types in assistive devices to augment the activation required for motor movements is believed to function by creating such an expectation of movement, decreasing beta power in the BG and motor cortex prior to movement onset.

To study this phenomenon, we had created a task that creates both expected and unexpected cue conditions. In the expected cue condition, we hypothesized and found a decrement in beta oscillatory activity after cue presentation, before movement onset. This is in keeping with the idea that an “expectation of movement” is required prior to movement onset, resulting in decreased beta activity in preparation for movement. In the unexpected −cue condition, we see that the decrement in beta power occurs later, in keeping with our prediction that there would be a lag associated with the self-generation of an internal cue to move. Once again, the internally generated cue sets in motion the preparatory decrease in beta activity prior to movement.

Our most interesting finding, however, is seen in the unexpected +cue condition. One may reasonably expect that in the presence of a cue, there would be a decrement in beta whether the cue is expected or unexpected. That is not what we hypothesized or found. Instead, beta remains present to a greater degree than in the expected +cue and the unexpected −cue conditions, after cue presentation. This is accompanied by greater movement time, as if the effort to move is handicapped by the unexpected nature of the cue. So what is going on?

We believe that the unexpected nature of the cue, in a PD environment, prevents the cell from adequately preparing for movement. We know that prior to movement onset, beta power in a normal subject decreases in the BG as well as motor cortex. This diminishment of beta in preparation for movement requires that the movement is anticipated. The lack of anticipatory capability in our experimental “unexpected +cue” condition prevents the beta decrease that necessarily precedes movement, while at the same time giving the system the “go cue” to move while the system is in an unprepared state. Without the ability to anticipate, in the “unexpected +cue” condition the preparatory decrease in beta does not occur normally.

Yet we have another unexpected condition, −cue, where beta did diminish appropriately prior to movement. The unexpected −cue condition differs from the unexpected +cue condition, however. Here, the expectation of movement was internally generated and movement preparation was carried out, thus creating a time lag to movement not seen in the “unexpected +cue” condition. Without a go cue, the system was free to first prepare for movement, and then generate an internal go cue that triggered movement that followed movement preparation. However when presented with an unexpected external go cue, no preparatory phase was possible. The movement in response to the go cue was, therefore, made hesitantly and haltingly, with beta activity present and movement time lengthening as a result of the lack of movement preparation.

What is the significance of this finding? We believe that we have found an experimental test condition that mimics the condition found in PD. In the Parkinsonian condition, there is an excess of beta oscillatory power. This abnormality is seen both in BG and motor cortex (Hutchison et al., 1994; Marsden et al., 2001; Brown, 2003; Gale et al., 2008; Hammond et al., 2007; Sarma et al., 2010). Movement in the PD patient occurs in the presence of greater beta than in normal subjects in STN and motor cortex. Our task strategy presented for the subject an unexpected cue for movement, providing no time for preparatory beta decrease. In a normal subject, our experimental condition may mimic PD by providing an elevated beta at the time of movement onset that would not be seen in a more typical scenario where environmental cues are expected.

However, these experiments were performed with Parkinsonian patients with existing abnormal beta dynamics. As a result, we see a worsening of existing beta that is additive to the already elevated beta seen in PD. Movement times are longer in PD patients, and in the “unexpected +cue” condition movement time was further extended. It would be interesting to see, outside the PD state in a normal subject, whether there would be excess beta after an unexpected cue. Stated alternatively, it is entirely possible that our ability to see this phenomenon results from abnormalities in movement preparation that is present only in the PD condition due to its abnormal PD dynamics.

What may be critical for motor facilitation in PD is a clear trigger that activates a pre-existing motor plan already formulated in prefrontal cortex. The mechanism of this activation would require the “expectation of movement” in the form of an expected external cue, or it can also be an internally generated cue (in our case generated in the absence of a visual cue) to move. In future work, we will record movement-related potentials for the same two-step task while we also record single unit neuronal activity in the STN to test the following hypothesis: *it is the activation of a specific motor plan, not the necessarily presentation of a cue, which is the critical event that provides the cortical drive that modulates the abnormal physiology of the basal ganglia, leading to motor facilitation in Parkinson's disease*. The importance here is that movement facilitation in PD does not derive from generalized cortical activation, or the activation of sensory cortical circuitry at any primary or associative level. This adds significant nuance to the neurophysiology of PD dysfunction that would be critical in future discussion of the disease. It would also be critical in devising potential future stimulation therapies, for example, that may be based upon finely tuned cortical activation.

### Conflict of interest statement

The authors declare that the research was conducted in the absence of any commercial or financial relationships that could be construed as a potential conflict of interest.
